# Elevated pulmonary arterial pressure in Zucker diabetic fatty rats

**DOI:** 10.1371/journal.pone.0211281

**Published:** 2019-01-28

**Authors:** Daniel Morales-Cano, Maria Callejo, Bianca Barreira, Gema Mondejar-Parreño, Sergio Esquivel-Ruiz, Sonia Ramos, María Ángeles Martín, Angel Cogolludo, Laura Moreno, Francisco Perez-Vizcaino

**Affiliations:** 1 Departament of Pharmacology and Toxicology, School of Medicine, Universidad Complutense de Madrid, Madrid, Spain; 2 Ciber Enfermedades Respiratorias (Ciberes), Madrid, Spain; 3 Instituto de Investigación Sanitaria Gregorio Marañón (IISGM), Madrid, Spain; 4 Department of Metabolism and Nutrition, Institute of Food Science and Technology and Nutrition (ICTAN), Consejo Superior de Investigaciones Científicas (CSIC), Madrid, Spain; 5 Ciber de Diabetes y Enfermedades Metabólicas Asociadas (CIBERDEM), Madrid, Spain; Max Delbruck Centrum fur Molekulare Medizin Berlin Buch, GERMANY

## Abstract

Diabetes is a very strong predictor of chronic systemic vascular diseases and acute cardiovascular events. Recently, associations between metabolic disorders and pulmonary hypertension have also been reported in both humans and animal models. In order to get some further insight into the relationship of pulmonary hypertension with obesity, insulin resistance and hyperglycemia, herein we have used the Zucker diabetic fatty rats (ZDF/*clr-lepr fa*) at 20 weeks fed a standard diet and compared to their lean Zucker littermates (ZL). ZDF rats were obese, had elevated plasma glucose levels and insulin resistance, i.e. a clinically relevant model of type 2 diabetes. They presented elevated systolic, diastolic and mean pulmonary arterial pressures and a parallel increase in the Fulton index. Systemic arterial pressures were also increased but the left ventricle plus septum weight was similar in both groups and the heart rate was reduced. Wall media thickening was observed in the small pulmonary arteries from the ZDF rats. Isolated pulmonary arteries mounted in a wire myograph showed similar vasoconstrictor responses to phenylephrine and 5-HT and similar responses to the endothelium-dependent vasodilator acetylcholine. However, the iNOS inhibitor 1400W enhanced the vasoconstrictor responses in ZDF but not in ZL rats. The protein expression of eNOS and iNOS was not significantly different in the lungs of the two groups. The lung expression of *Bmpr2* mRNA was downregulated. However, the mRNA expression of *Kcna5*, *Kcnk3*, *Kcnq1*, *Kcnq4* or *Kcnq5*, which encode for the potassium channels Kv1.5, TASK-1, Kv7.1, Kv7.4 and Kv7.5, respectively, was similar in ZL and ZDF rats. In conclusion, ZDF rats show increased pulmonary arterial pressure, right ventricular hypertrophy, pulmonary arterial medial thickening and downregulated lung *Bmpr2* despite leptin resistance. These changes were mild but are consistent with the view that diabetes is a risk factor for pulmonary hypertension.

## Introduction

Pulmonary hypertension (PH) is characterized by an increase in pulmonary arterial pressure (PAP) and pulmonary vascular resistance determined by right heart catheterization at rest [1]. It is classified into 5 groups according to its origin: 1) pulmonary arterial hypertension (PAH), 2) PH hypertension associated with left heart disease, 3) PH associated with lung diseases and hypoxia, 4) PH associated to chronic thromboembolism (CTEPH), and 5) PH of unknown origin or multifactorial [1]. With the exception of idiopathic PAH, in all groups and subgroups of PH there is a known factor, such as a mutation, infection, hypoxia, drugs, embolism or other diseases, that is associated with the development of the disease. However, none of these factors by itself is sufficient to trigger the disease [2]. The clinical risk factors that predict the development of PH in patients at risk, i.e. the so called “second hits”, have not yet been fully identified.

In recent years there has been a worldwide increase in the prevalence of type 2 diabetes [3], which is a very well-known predictor of chronic systemic vascular diseases and acute cardiovascular events [4]. Recently, associations between metabolic disorders and pulmonary hypertension have also been reported [5]. Several studies have suggested that insulin resistance and type 2 diabetes are associated with pulmonary hypertension in humans [6–9]. However, the relation of PH with obesity, which is very frequently associated to insulin resistance and diabetes, is unclear. Systolic PAP has been positively correlated with body mass index in 3790 echocardiographically normal subjects [10]. Paradoxically, obesity has also been suggested as a protective prognostic factor in patients with PH [11].

Several studies in rodents have also shown pulmonary vascular dysfunction in diabetes. Type 1 diabetic animals show pulmonary endothelial dysfunction, BMPR2 downregulation and lung inflammation [12, 13]. These factors alone are insufficient to increase pulmonary arterial pressure but potentiate the effect of hypoxia [14]. The insulin resistant ApoE knockout mice fed on a high fat diet, which have increased blood glucose levels but normal or moderately increased body weight, show PA remodeling and increased PAP [15]. In contrast, the obese non-diabetic Zucker model (OZR), characterized by a mutation in the leptin receptor yielding high circulating leptin levels, obesity and insulin resistance but normal fasting blood glucose, does not present any of the characteristic features of pulmonary vascular disease but rather a hyporresponsiveness to several pulmonary vasoconstrictors [16]. However, at Denver’s altitude, in OZR, overfeeding elicited PA remodeling, neomuscularization of distal arterioles, and elevated PA pressure, accompanied by right ventricular hypertrophy [17]. The Zucker diabetic fatty rats (ZDF/ *crl-lepr fa*) is an inbred strain derived from OZR carrying an additional yet unidentified mutation leading to hyperglycemia after 10–12 weeks of age [18–20]. At 4 weeks of age, ZDF rats with moderate weight gain, normoglycemia and normoinsulinemia not only had normal PAP and right ventricular weight but also were partially protected from hypoxia-induced PH [21].

In order to get some further insight into the relationship of PH with obesity, insulin resistance, leptin and hyperglycemia, the present study was designed to analyze the key features of the pulmonary circulation in ZDF at 20 weeks of age, a rat model of obesity, hyperlipidemia, insulin resistance and hyperglycemia, i.e. a clinically relevant model for type 2 diabetes in humans.

## Materials and methods

### Animals and ethics statement

All experimental procedures utilizing animals were carried out according to the Spanish Royal Decree 1201/2005 and 53/2013 on the Care and Use of Laboratory Animals and approved by the institutional Ethical Committees of the Universidad Complutense de Madrid (Madrid, Spain) and the regional Committee for Laboratory Animals Welfare (Comunidad de Madrid, Ref. number PROEX-304-15). Male ZDF rats (ZDF/*crl-lepr fa*, n = 6) and their lean littermates (ZL, +/?, n = 6) were obtained from Charles River Laboratories (L'Arbresle, France). Animals were maintained on standard chow diet and weekly monitored for blood glucose and body weight.

### Hemodynamic measurements and right ventricular hypertrophy

Rats were anesthetized (80 mg/kg ketamine and 8 mg/kg xylacine i.p.), tracheostomyzed and ventilated with room air (tidal volume 9 mL/kg, 60 breaths/min, and a positive end-expiratory pressure of 2 cm H_2_O, Nemi Scientific Inc, Medway, USA). Systolic, diastolic and mean systemic arterial pressure (sSAP, dSAP and mSAP) was measured by cannulation of the carotid artery in closed chest animals. Then, after sternotomy, a catheter was placed in the pulmonary artery (PA) through the right ventricle for systolic, diastolic and mean PA pressure (sPAP, dPAP and mPAP) recording [22]. It should be noted that open-chest measurements in anaesthetized animals underestimate real PAP. We estimated oxygen consumption by the rate pressure product as the product of heart rate and systolic pressure multiplied by 10^−3^ at systemic and pulmonary levels [23, 24], and pulse pressure was defined as the difference between systolic and diastolic arterial pressure. At the end of the experiment, the right ventricle (RV) and the left ventricle plus the septum (LV+S) were dissected and weighed. The Fulton Index [RV/(LV+S)] was calculated to assess the right ventricular hypertrophy.

### Lung histology

The right lung was inflated in situ with formol saline through the right bronchus and embedded in paraffin. Lung sections were stained with haematoxylin and eosin and examined by light microscopy, and elastin was visualized by its green auto-fluorescence. Small arteries (25–100 mm outer diameter) were analyzed in a blinded fashion and categorized as muscular, partially muscular or non-muscular as previously described [22]. Media thickness was measured using image-J software. Four to eight photographs were taken and at least eight arteries were analyzed from each animal.

### Vascular reactivity

Resistance PA rings (diameter ~0.3–0.5 mm and length ~2 mm) were mounted in Krebs solution at 37°C gassed with a 21% O_2_-5% CO_2_ mixture in a wire myograph. After stretching to give an appropriate resting tension (equivalent to 30 mm Hg) vessels were sequentially exposed to different vasoconstrictor agents to test the vascular response, KCl (80 mmol/L), phenylephrine (Phe, 1 nmol/L-10 μmol/L) and serotonin (5-HT, 30 nmol/L-30 μmol/L). The contractile responses were performed by cumulative drug addition and the tissues were washed 3 times with drug-free Krebs after each stimulus and allowed to recover for 30 min. The endothelial function was estimated by the analysis of the relaxant response to the cumulative addition of acetylcholine (1 nmol/L-10 μmol/L) after precontraction with a concentration of phenylephrine titrated to induce a contraction 75% of the response to KCl. Some experiments were carried out in the presence of the inducible nitric oxide synthase inhibitor iNOS inhibitor 1400W (10 μmol/L).

### RNA extraction and quantitative RT-PCR

Total RNA was extracted from lung tissue using miRNeasy Mini Kit (Qiagen, Hilden, Germany) in accordance with the manufacturer’s instructions. RNA concentration and quality was checked using NanoDropTM 1000 Spectrophotometer (Thermo Scientific). One μg of RNA was reverse transcribed into cDNA using iScriptTM cDNA Synthesis Kit (Biorad, California, USA) following manufacturer’s instructions. Gene expression was determined in triplicates by quantitative real-time PCR (qRT-PCR) with a Taqman Gene Expression Master Mix (Ref: 4369016, Applied Biosystems), with specific primers from Applied Biosystems (Table 1) in the Genomic Unit of the Universidad Complutense de Madrid. A standard Taqman amplification protocol was used as follows: 10 min at 95°C followed by 40 cycles of 15 s at 95°C (denaturation) and 1 min at 60°C (annealing). The efficiency was calculated in preliminary experiments by serial sample dilutions. The delta-delta Ct method was used to quantify relative changes. mRNA expression was normalized by the geometrical mean of the expression of β-actin and β2-microglobulin. [25]

**Table 1:** Taqman primers used in this study (all from Applied Biosystems).

Gen	Reference	Gene Bank	Amplicon lenght	R^2^	Efficiency
Actin, beta (*Actb*)	Rn00667869_m1	NM_031144.3	91	0.99	87%
Beta-2 microglobulin (*B2m*)	Rn00560865_m1	NM_012512.2	58	0.99	103%
Bone morphogenetic protein receptor type 2 (*Bmpr2*)	Rn01437214_m1	NM_080407.1	104	0.99	108%
Potassium two pore domain channel subfamily K member 3 (*Kcnk3*)	Rn04223042_m1	NM_033376.1	72	0.99	99%
Potassium voltage-gated channel subfamily A member 5 *(Kcna5)*	Rn00564245_s1	NM_012972.1	63	0.99	102%
Potassium voltage-gated channel subfamily Q member 1 *(Kcnq1)*	Rn00583376_m1	NM_032073.1	85	0.99	97%
Potassium voltage-gated channel subfamily Q member 4 *(Kcnq4)*	Rn01518851_m1	XM_008764109.1	71	0.99	102%
Potassium voltage-gated channel subfamily Q member 5 *(Kcnq5)*	Rn01512013_m1	NM_001134643.2	89	0.99	102%

### Protein expression

Whole lungs were homogenized with a lysis buffer (Trizma Pre-set cystals pH 7.5, DL-dithiothreitol (DTT, 1M), NP40 (1%) and supplemented with protease (Protease inhibitor cocktail tablets, Roche Diagnosis GmbH) and phosphatase (PhosSTOP, Roche Diagnostics GmbH) inhibitors in a Tissuelyser device (Qiagen, Hilden, Germany). After four short pulses (30 seconds, stopping 15 seconds between each pulse) of sonication, the lysates were centrifuged for 10 min at 10000 rpm. Protein concentration was determined by a colorimetric assay based on the Lowry method (Biorad, California, USA). Twenty μg for lung homogenates were run on a sodium dodecyl sulphate-polyacrilamide electrophoresis and proteins were transferred to polyvinylidene difluoride membranes (Biorad, California, USA). Membranes were blocked by incubation with 5% of BSA or milk for one hour, and were incubated overnight at 4°C with primary antibodies (Table 2). Membranes were then incubated with the appropriate secondary antibodies conjugated with horseradish peroxidase at room temperature for one hour. Antibody binding was detected by an ECL system (SuperSignal West Fento Chemiluminescent Substrate, Thermo Scientific, USA). Blots were imaged using an Odissey Fc System (Li-COR Biosciences, USA) and were quantified by densitometry using Quantity One software. Results were normalized to the relative expression of smooth muscle β-actin.

**Table 2:** Primary antibodies used in this study.

Primary antibody	Protein	Specie	Supplier	Reference	Dilution
Anti-β-Actin	β-actin	Mouse	Sigma-Aldrich	A1978	1:5000
Anti-eNOS	eNOS	Mouse	BD Transduction Laboratories	610296	1:1000
Anti-iNOS	iNOS	Rabbit	Santa Cruz Biotechnology	SC-650	1:200

### Measurement of proinflammatory cytokines

Tumor necrosis factor-α (TNF-α) and interleukin-6 (IL-6) levels were quantified in serum samples and lung homogenates by specific rat TNF-alpha Quantikine ELISA Kit (RTA00, R&D System, USA) and IL-6 DuoSet ELISA Kit (DY506, R&D System, USA) according to the manufacturer’s instruction.

### Drugs

All drugs were from Sigma-Aldrich Quimica (Spain).

### Statistical analysis

Data are expressed as means ± s.e.m. All data passed normality test. Statistical comparisons were performed, unless otherwise stated, using two-tailed unpaired t tests for continuous variables. The Chi square test was used for the analysis of arterial muscularization. P < 0.05 was considered statistically significant.

## Results

### Body weight, glucose, insulin and cytokines

At the age of 20 weeks, ZDF showed marked increases in body weight, plasma glucose and plasma insulin as compared to ZL (Table 3, P < 0.01 for all comparisons). As expected, insulin resistance, measured by the HOMA-IR index, was significantly increased in ZDF rats compared to lean controls (P < 0.01). The circulating levels of the cytokines IL-6 and TNF-alpha in serum or lung IL-6 were not significantly different between ZL and ZDF rats (Table 3).

**Table 3:** Body weight, plasma glucose, insulin, HOMA-IR index and cytokine levels.

	Body weight (g)	Plasma glucose (mg/dL)	Insulin (μg/L)	Homa-IR index	Serum IL-6 (pg/ml)	Serum TNFα (pg/ml)	Lung IL-6 (ng/mg protein)
ZL	328 ± 6	79 ± 5	0.38 ± 0.02	2.7 ± 0.1	166 ± 20	13 ± 1	1.67 ± 0.28
ZDF	455 ± 11 **	262 ± 17 **	4.50 ± 0.50 **	87 ± 11 **	197 ± 87	13 ± 1	1.40 ± 0.33

Values are expressed as means ± s.e.m. n = 6 per group.

** indicates P < 0.01, ZDF versus ZL (unpaired t test).

### Hemodynamics and RV hypertrophy

**Fig 1:**
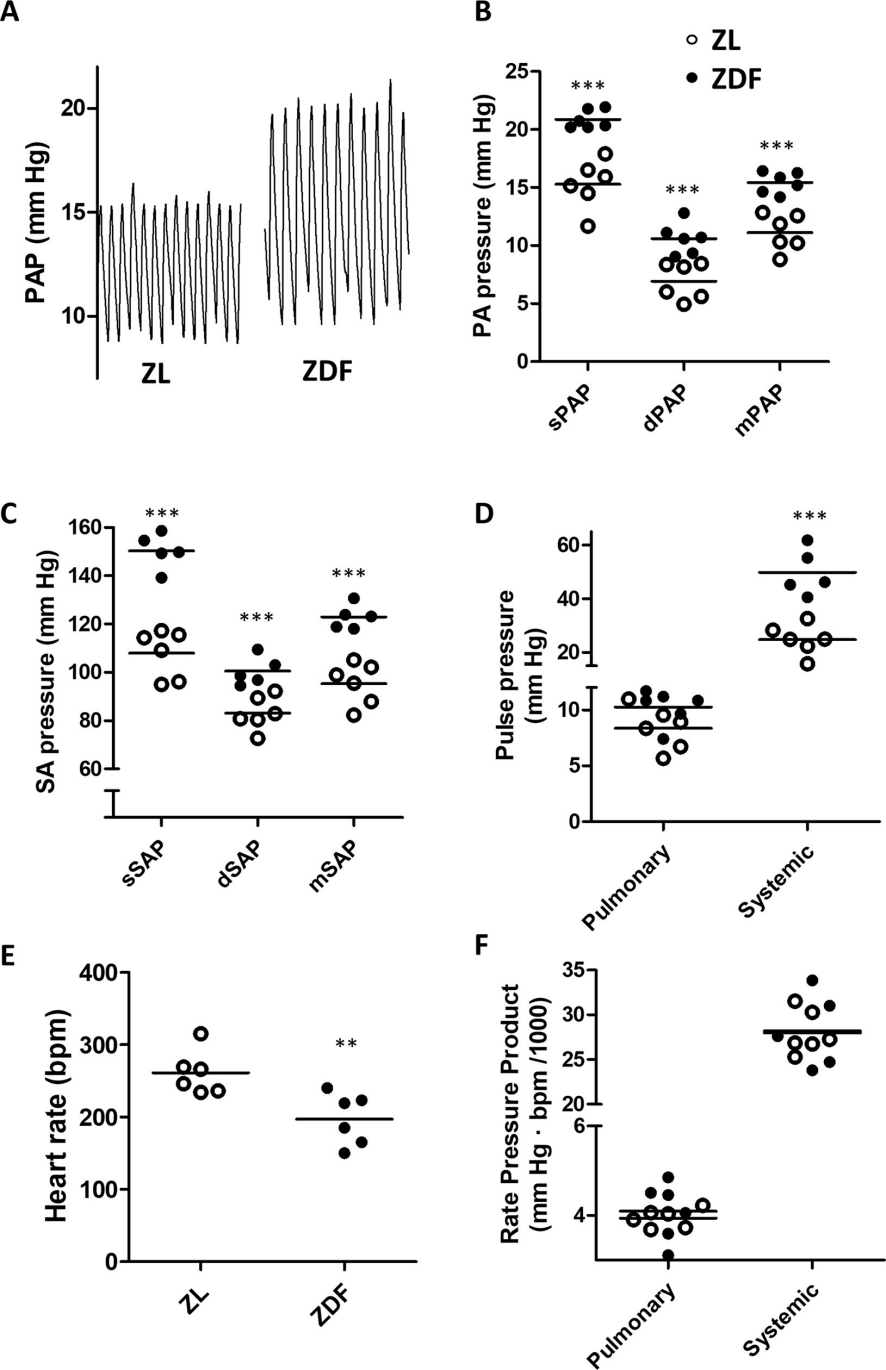
ZDF rats show increased pulmonary and systemic arterial pressure. (A) Typical pulmonary arterial pressure (PAP) recordings. (B) Systolic, diastolic and mean PAP. (C) Systolic, diastolic, and mean systemic arterial pressure (SAP). (D) Pulse pressure, (E) Heart rate, (F) Rate pressure product. Data are shown as scatterplots and means of 6 animals (except SAP could not be recorded in one ZDF rat). ** and *** indicate P < 0.01 and P< 0.001, respectively, ZDF versus ZL (unpaired t test).

**Fig 2:**
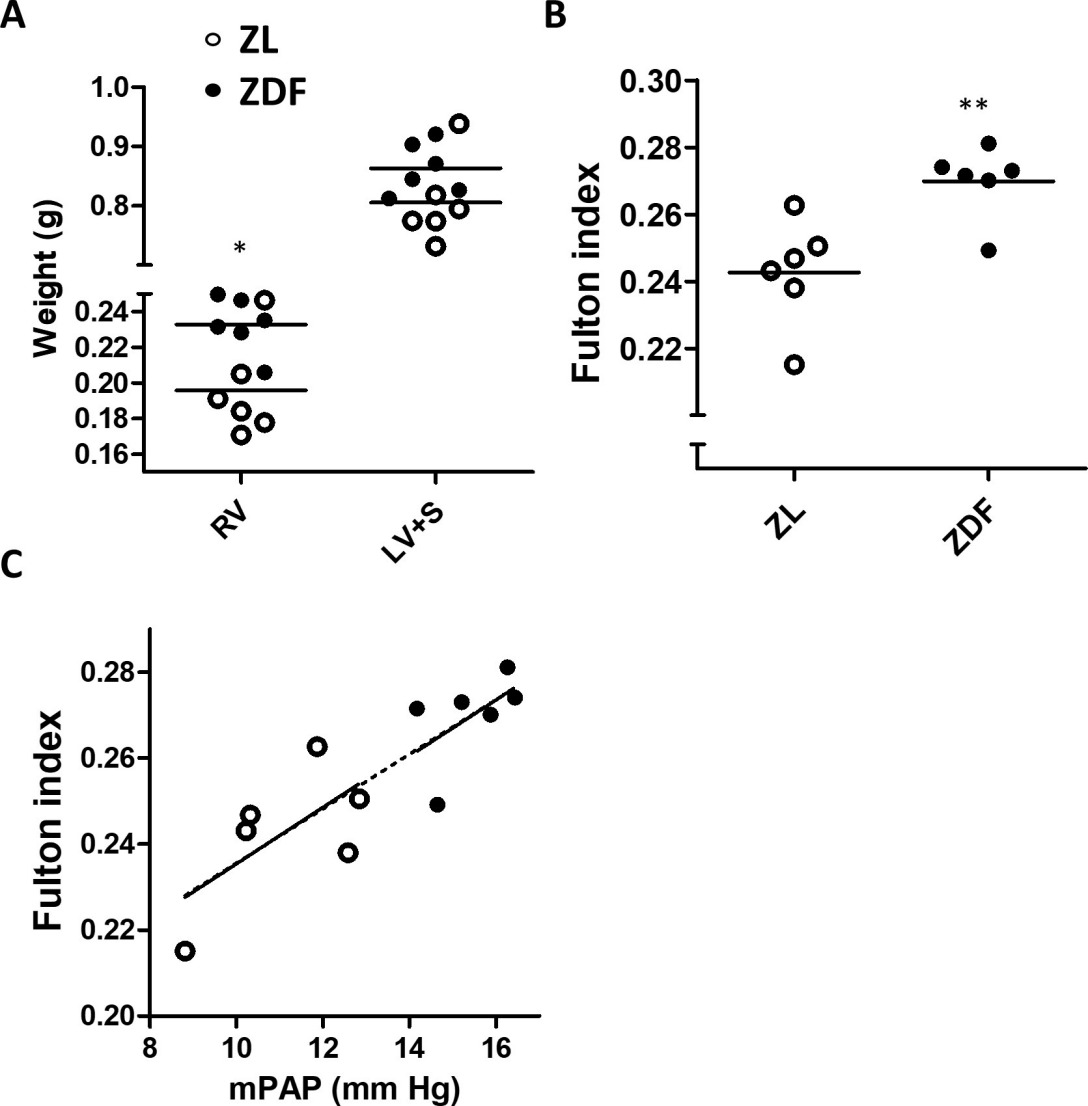
ZDF rats show right ventricular hypertrophy. (A) Right ventricular (RV) weight and left ventricular plus septum (LV+S) weight as absolute values and (B) Fulton index [RV/(LV+S) ratio]. (C) Correlation between mPAP and Fulton index. The dotted line represents the linear regression for pooled data from both groups (r^2^ = 0.72, p< 0.001. Results are expressed as scatter plots and means of 6 animals, *, ** and *** indicate P < 0.05, P < 0.01 and P< 0.001, respectively, ZDF versus ZL (unpaired t test).

The systolic, diastolic and mean PAP were significantly elevated in ZDF when compared with ZL rats (Fig 1A and 1B). This was accompanied by increased systolic, diastolic and mean systemic arterial pressures (SAP, Fig 1C). Differences were more marked for systolic than for diastolic pressures and, therefore, pulse pressure was significantly elevated in the systemic but not in in the pulmonary circulation (Fig 1D). ZDF rats also showed decreased heart rate (Fig 1E). The rate pressure product as an indicator of the myocardial oxygen consumption did not show differences between ZL and ZDF rats at systemic and pulmonary levels (Fig 1F). Absolute RV but not LV+S weight was significantly increased in ZDF rats (Fig 2A). Notably, the RV weight referred to LV+S, i.e. the Fulton index, was also elevated in ZDF rats (Fig 2B). RV contractility estimated by (dP/dt max)/P calculated from the right ventricular pressure wave was not different in the two groups (37.6 ± 1.5 *vs* 35.2 ± 1.2 s^-1^, respectively). Fig 2C shows that there is a good correlation between the mPAP and the Fulton index, which was consistent when data from both groups of rats were analyzed individually or when all values were pooled.

### Lung histology

**Fig 3:**
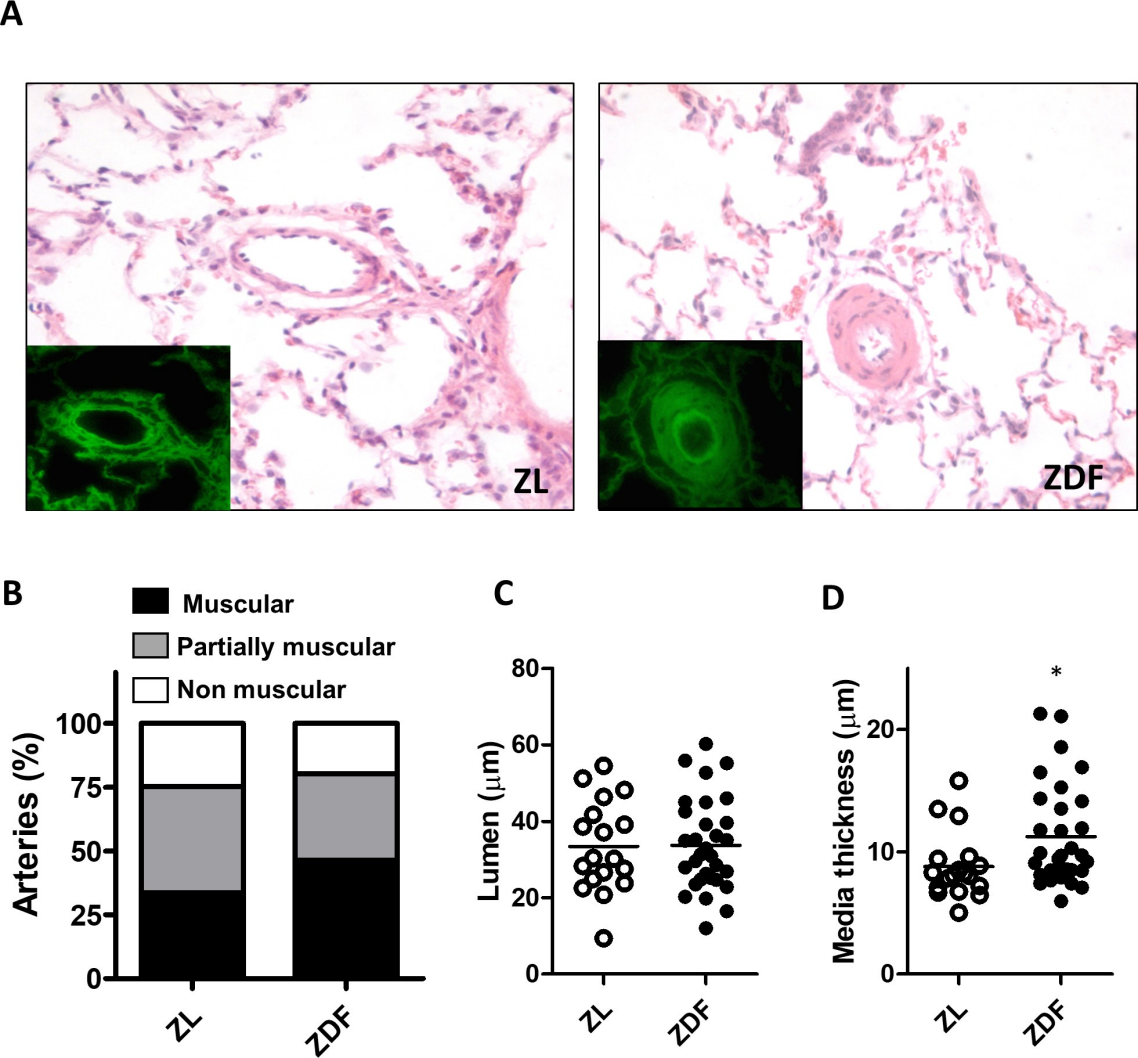
Histological changes in the pulmonary circulation. (A) Representative images of cross-sections of lungs stained with hematoxylin and eosin. On the left a non muscularized small PA from a ZL rat. On the right a muscularized small PA from a ZDF rat. The insets show the green autofluorescence of elastin. (B) Percentage of muscular, partially muscular and non-muscular PA. (C) Lumen and (D) Media wall thickness in PA < 75μm. Results are expressed as scatter plots of media thickness in each individual artery from 6 ZL and 6 ZDF rats. * indicates P < 0.05, ZDF versus ZL (unpaired t test).

Small PA from lung sections from ZL and ZDF rats (Fig 3A) were classified in a blinded fashion as muscular, partially muscular and non-muscular arteries. The percentage of muscularized arteries was not significantly different (Fig 3B) in ZL vs ZDF. However, the small muscularized arteries in ZDF rats of lumen less than 75 μm (Fig 3C) showed a modest but significant increase in pulmonary arterial wall thickness compared to ZL rats (Fig 3D, P<0.05). We did not observe other apparent histological changes.

### Vascular reactivity

**Fig 4:**
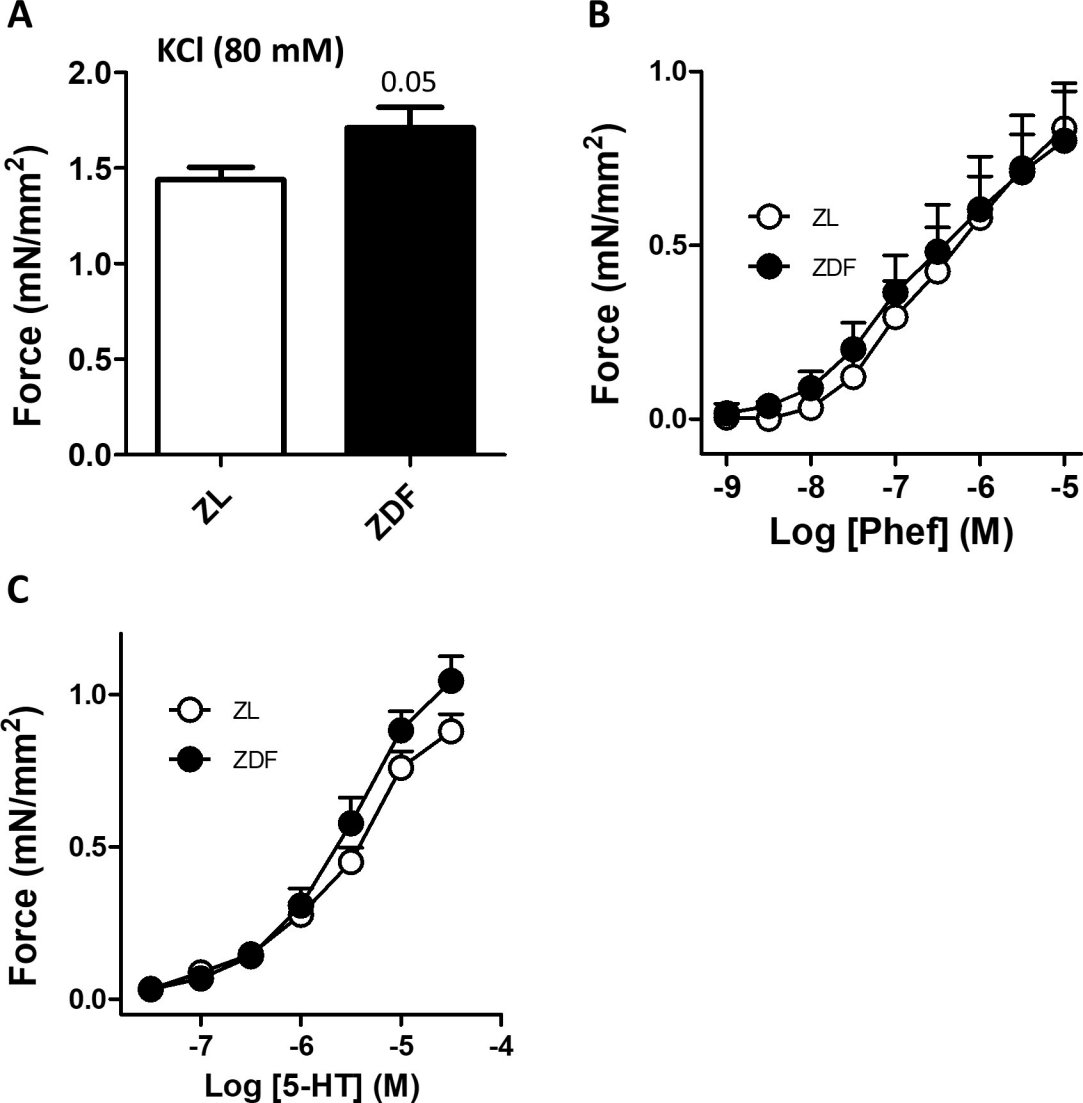
ZDF rats show normal PA pulmonary artery contraction. (A) Contractile responses to 80 mM KCl. (B and C) Cumulative concentration-response curves to (B) the alpha-adrenergic agonist phenylephrine and (C) serotonin (5-HT). Results are expressed as means ± SEM of 5 (ZDF) and 4 (ZL) rats in duplicate. P = 0.05, ZDF versus ZL (unpaired t test).

**Fig 5:**
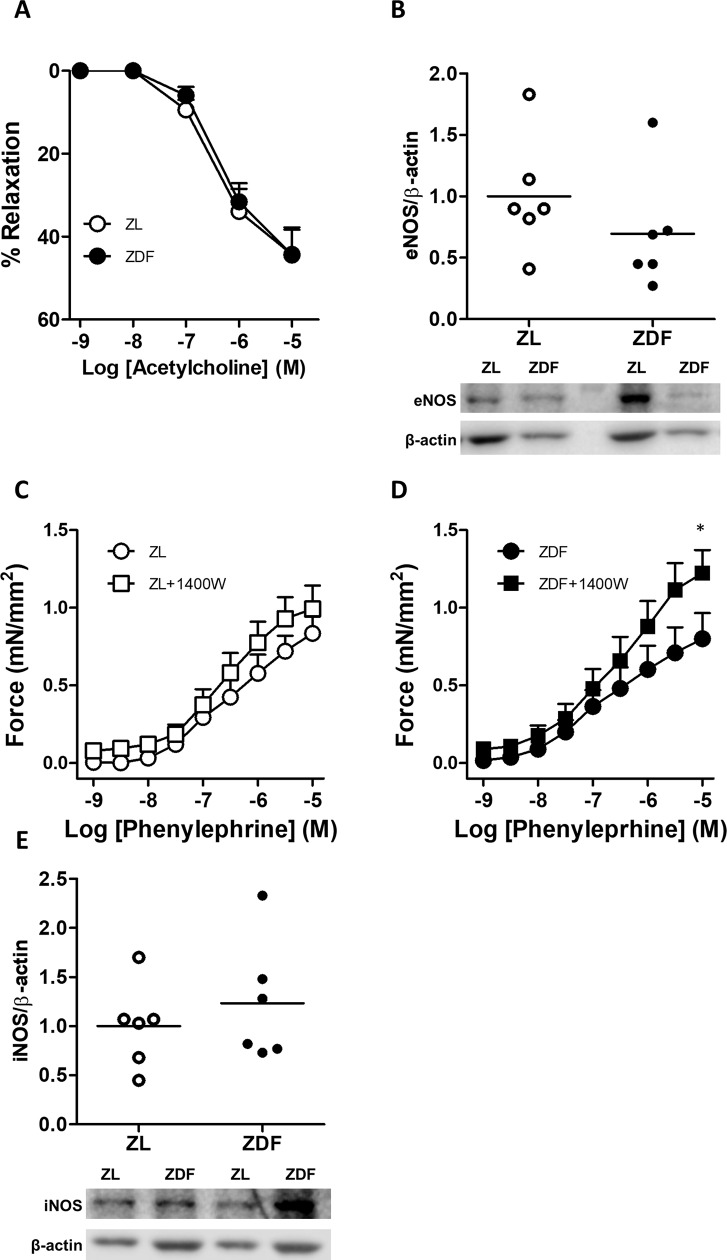
ZDF rats show normal PA pulmonary artery endothelial function and eNOS expression but iNOS inhibition unmasks vasoconstrictor hyperresponsiveness in ZDF rats. (A) Cumulative concentration-response curves to the endothelium-dependent vasodilator acetylcholine in arteries precontracted with phenylephrine. (B) Expression of endothelial NOS (eNOS) analyzed by Western Blot, typical blots (bottom panel) and densitometric values (top panel). (C and D) Cumulative concentration-response curves to phenylephrine (Phef) in the absence or in the presence of the iNOS inhibitor 1400W (10 μmol/L). (E) Expression of inducible NOS (iNOS) analyzed by Western Blot, typical blots (bottom panel) and densitometric values (top panel). Results for vascular reactivity are expressed as means ± SEM of 5 (ZDF) and 4 (ZL) rats in duplicate. * indicates P < 0.05 ZDF versus ZDF+1400W (paired t test). Results for protein expression are expressed as scatter plots and means of 6 animals normalized by the expression of β-actin.

The responses to vasoconstrictor and vasodilators were analyzed in isolated PA mounted in a wire myograph (Figs 4 and 5). The response to KCl, which is regarded as an index of the contractile capacity of the vessel, was borderline significantly increased in ZDF rats (Fig 4A). However, we found no differences in the concentration-response curves for the vasoconstrictor responses to the α-adrenergic receptor phenylephrine (Fig 4B) or to serotonin (5-HT, Fig 4C) in ZDF compared to ZL. The endothelium-dependent vasodilator responses to acetylcholine (Fig 5A) were also similar in the two groups. Consistently, the protein expression of eNOS in the lung was also similar (Fig 5B). We also analyzed the effect of the iNOS inhibitor 1400W on the contractile responses induced by phenylephrine in ZL (Fig 5C) and ZDF (Fig 5D) rats. This inhibitor modestly increased the response to the vasoconstrictor in ZDF which was only significant at the highest concentration analyzed (paired t test) but had no significant effect in the ZL rats. However, iNOS expression in whole lung homogenates was not significantly different between groups (Fig 5E).

### Gene expression

**Fig 6:**
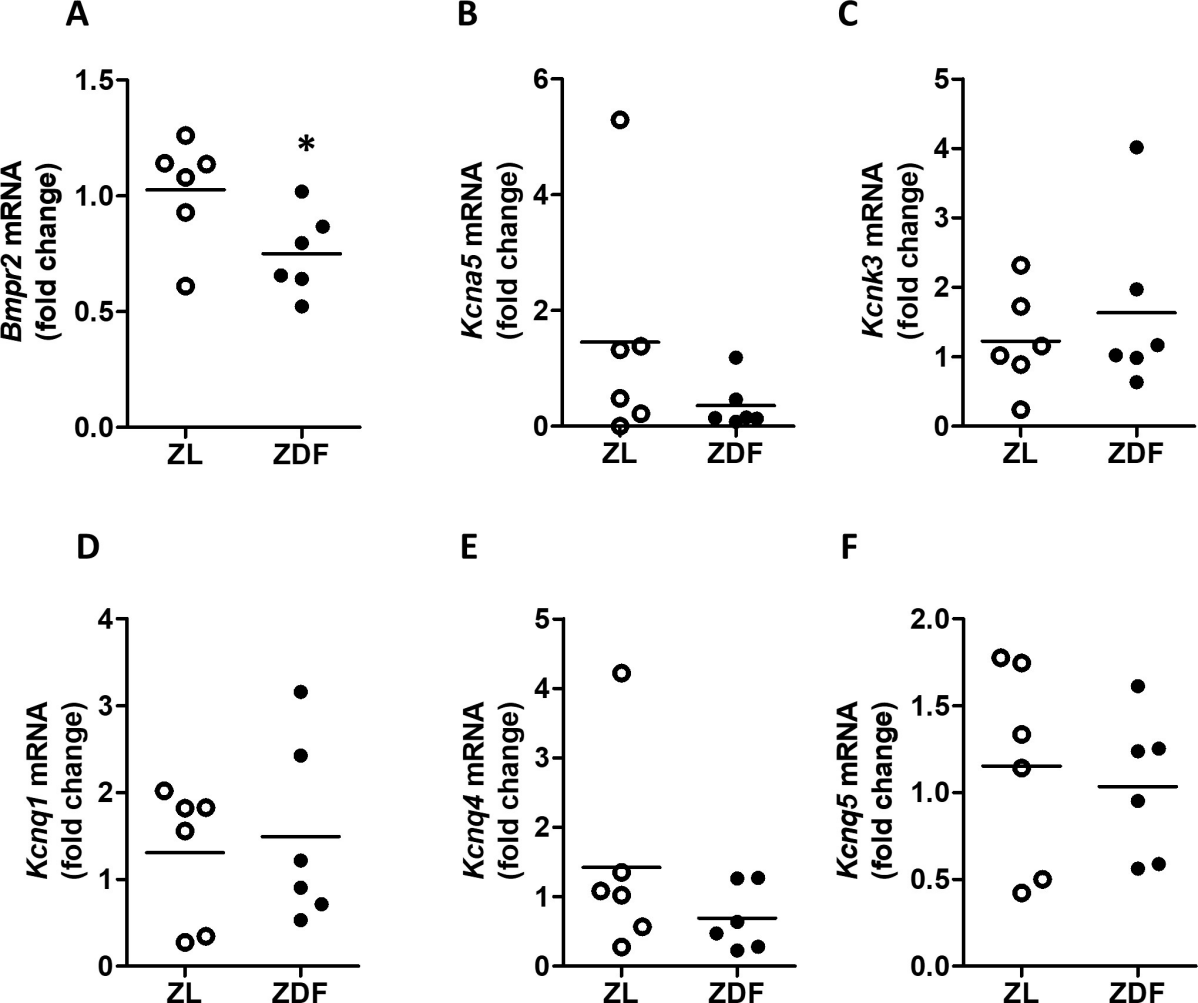
**Changes in mRNA expression of (A) *Bmpr2*, (B) *Kcna5*, (C) *Knck3*, (D) *Kcnq1*, (E) *Kcnq4* and (F) *Kcnq5* mRNA.** Expression was analyzed by qRT-PCR. Results are scatter plots and means of 6 animals normalized by the expression of β-actin and β2-microglobulin. Results are expressed as scatter plots and means of 6 animals. * indicates P < 0.05 ZDF versus ZL (unpaired t test).

We analyzed the expression of several genes involved in PH such as *Bmpr2*, *Kcna5*, *Knck3*, *Kcnq1*, *Kcnq4* and *Kcnq5* in the lungs of ZL and ZDF rats (Fig 6). *Bmpr2* (Fig 6A) was significantly reduced in ZDF rats but the other genes were unchanged. There was a trend for reduced *Kcna5*, which encodes the voltage-gated potassium channel Kv1.5, but the expression was highly variable and differences were not statistically significant (Fig 6B). The expression of *Kcnk3*, which encodes the voltage-independent channel TASK-1, was similar in the two groups (Fig 6C). In addition, *Kcnq1*, *Kcnq4* and *Kcnq5*, which encode the voltage-dependent channels Kv7.1, Kv7.4 and Kv7.5, were also not significantly different (Fig 6D and 6E).

## Discussion

The results of the present study indicate that when compared to their lean littermates, ZDF rats show elevated PAP, RV hypertrophy, remodeling of the vessel structure and altered pattern of lung gene expression. All these changes were mild. Consistent with previous reports [26, 27] these animals also showed elevated systemic arterial pressure. However, there was no apparent change in the responses to vasoconstrictors in the PA, except that the responses were mildly potentiated in ZDF rats by the iNOS inhibitor 1400W. In addition, ZDF rats did not show apparent pulmonary endothelial dysfunction as indicated by a response to the endothelial-dependent vasodilator acetylcholine similar to that of ZL rats.

The mechanism linking metabolic defects to PAH is not clear. Herein, we describe the major features of the pulmonary circulation in a rat model of obesity, hyperinsulinemia and hyperglycemia. The lean littermates are used for internal comparison. In addition, we are also comparing our present results with previously published models of hyperglycemia without obesity and without hyperinsulinemia (streptozotocin-induced type 1 diabetes), with obesity and hyperinsulinemia without hyperglycemia (obese Zucker rats) and with obesity without hyperinsulinemia and without hyperglycemia (diet-induced obesity and young ZDF rats) from our group and others.

Previous studies have shown that either hyperglycemia or insulin-resistance are able to induce an increase in PAP only when combined with moderate normobaric hypoxia or hypobaric hypoxia associated to altitude [13, 14, 16, 17]. Herein, however, we show that hyperglycemia plus insulin resistance, i.e. frank type 2 diabetes, induces an increase in PAP even at low altitude. The changes in RV weight, relative to the LV+S weight (i.e. the Fulton index) paralleled those in mPAP. Moreover, we found a good correlation between those two parameters, supporting that RV hypertrophy in diabetes is a direct consequence of elevated PAP.

PH is consistently associated to the structural remodeling of the pulmonary vascular tree with increased muscularization playing a pathophysiological role in the increase in pulmonary vascular resistance. Herein, we found a modest increase in the arterial wall thickness. It should be noted that remodeling may precede the increase in PAP in metabolic disease because remodeling may be present in the absence of increased PAP as observed in the type 1 diabetic animals under normoxia [14].

Animal models of diabetes and insulin resistance also support a link between pulmonary vascular dysfunction and altered glucose metabolism. Endothelial dysfunction, typically measured by the relaxant response to acetylcholine, and hyperresponsiveness to 5-HT-induced vasoconstriction are hallmarks of PH in animal models. Pulmonary vascular function has been studied in rats treated with streptozotocin as a type 1 diabetes model, i.e. insulinopenic, hyperglycemic and lean rats. Our group showed a marked endothelial dysfunction in PA characterized by an increase of reactive oxygen species and by an increased expression of p47phox [12, 14] which is consistent with results in most systemic vascular beds. OZR rats, which show obesity and insulin resistance but are normoglycemic, had normal endothelial function in both large and small PA [16]. Contrary to our expectations, in the present study, the similar relaxant response to acetylcholine and unchanged lung eNOS expression revealed a preserved endothelial function in ZDF rats despite a large increase in blood glucose. Similarly, hyperresponsiveness to 5-HT was found in hyperglycemic, lean streptozotocin-treated rats [12, 14] but it was absent in either normoglycemic OZR [16] or ZDF rats (present results). Therefore, these results might be consistent with obesity being a protective mechanism for vascular function.

Both PAH and obesity are regarded as inflammatory conditions associated with induction of cytokines (e.g. IL-6 and TNF-α) and pro-inflammatory enzymes (e.g. iNOS). In PA from OZR we found increased iNOS protein expression and increased response to the iNOS inhibitor 1400W [16]. In the ZDF the response to 1400W was also mildly increased. However, iNOS expression was not statistically different, possibly because it was analyzed in the whole lung rather than in isolated PA. In addition, we found no significant differences between ZL and ZDF rats in the lung or serum IL-6 or serum TNF-α, which is consistent with previous data in the lungs and other organs in OZR and ZDF rats at this age and fed with standard diet [28–30]. However, increased circulating levels of these pro-inflammatory cytokines is usually observed in older animals or animals fed a high fat diet [30, 31]. Because IL-6 deficient mice are partially protected from pulmonary hypertensive stimuli [32], high fat fed animals with increased cytokine levels might possibly show larger changes in vascular dysfunction and PAP than those observed herein.

We analyzed the expression of several genes, which are found to be mutated in some patients with heritable PAH or downregulated in several forms of non-heritable human and experimental PH [2], e.g. *Bmpr2*, *Kcna5* and *Knck3*, which encode for the BMPR2, KV1.5 and TASK1 proteins, respectively. The activity of BMPR2 exerts an antiproliferative effect in PA smooth muscle cells and its defect is thought to play a major role in arterial remodeling associated to PH [2]. The downregulation of *Bmpr2* mRNA may account for the increased wall thickening of the PA that we found in the ZDF rat lungs. Similarly, downregulated lung *Bmpr2* has also been found in a rat model of type 1 diabetes but not in the insulin-resistant OZR. In addition, reduced *Bmpr2* has been found in the kidney of type-1 diabetic rats [33]. These results suggest that hyperglycemia, but not insulin-resistance or obesity, is associated to decreased *Bmpr2* expression. Moreover, paradoxically, increased BMPR2 expression has been reported in the adipose tissue and has been suggested to play a role in the pathophysiology of obesity [34].

Kv1.5 channels are responsible for the major component of the voltage-dependent potassium currents in pulmonary arteries and its downregulation in PH contributes to increased PA depolarization and contraction [35]. Its expression is unchanged in type-1 diabetes [13] or in insulin-resistant OZR [16]. In the ZDF there was large variability in the channel expression but overall there was no statistical change. The loss of *Kcnk3* expression and activity is also a key event in PAH pathogenesis [36]. However, the expression of Kcnk3 was unchanged in ZDF rats.

We also analyzed *Kcnq1*, *Kcnq4* and *Kcnq5*, which encode for Kv7.1, Kv7.4 and Kv7.5, respectively, because they are involved in the regulation of pulmonary vascular tone and because they have been shown to be downregulated in coronary arteries from diabetic animals [37]. Contrary to our expectations these genes were similarly expressed in ZL and ZDF rats. However, this may reflect the different expression and regulation of Kv7 channels in different tissues [38].

The limitations of the present study that should be mentioned include: 1) the hemodynamic study was conducted in open chest ventilated rats under anesthesia, 2) Part of the data on expression were analyzed by RT-PCR only (not at the protein level).

Paradoxically, obesity has also been suggested as a protective prognostic factor in patients with PH [11]. Reduced sensitivity to leptin, an adipokine reported to have a pathophysiological role in PH [21, 39], in obese patients may account for the protective effect of obesity. In fact, young ZDF were partially protected from hypoxia-induced PH [21]. Our results in adult ZDF rats indicate that reduced leptin sensitivity is not sufficient to prevent hyperinsulinemia- and hyperglycemia-induced pulmonary vascular dysfunction.

## Conclusions

Previous models of hyperglycemia or obesity with or without hyperinsulinemia have shown different anatomic or functional PA alterations. These animals developed increased PAP and RV hypertrophy only when combined with moderate altitude or normobaric hypoxia. Herein, we report that the ZDF rat which develops frank type 2 diabetes with hyperglycemia, hyperinsulinemia and obesity at 20 weeks of age shows elevated PAP, PA remodeling, RV hypertrophy and *bmpr2* downregulation making this animal a suitable model to study the association between glucose and lipid metabolic dysregulation and pulmonary hypertension. The present data also support the continuous growing evidence that diabetes is a risk factor for PH.

## Supporting information

S1 File Raw data. (XLSX)
